# Autoinhibition of the GEF activity of cytoskeletal regulatory protein Trio is disrupted in neurodevelopmental disorder-related genetic variants

**DOI:** 10.1016/j.jbc.2022.102361

**Published:** 2022-08-10

**Authors:** Josie E. Bircher, Ellen E. Corcoran, TuKiet T. Lam, Michael J. Trnka, Anthony J. Koleske

**Affiliations:** 1 Department of Molecular Biophysics and Biochemistry, Yale University, New Haven, Connecticut, USA; 2 Keck MS & Proteomics Resource, Yale University, New Haven, Connecticut, USA; 3 Department of Pharmaceutical Chemistry, University of California at San Francisco, San Francisco, California, USA; 4 Department of Neuroscience, Yale University, New Haven, Connecticut, USA

**Keywords:** GEF, Rho GTPase, neurodevelopmental disorder, Trio family proteins, spectrin repeats, autoinhibition, ADB, antibody dilution buffer, BS3, bis(sulfosuccinimidyl)suberate, DH1, Dbl homology domain, FL, fluorescein, GEF, guanine exchange factor, NDD, neurodevelopmental disorder, Ni-NTA, nitrilotriacetic acid, PH1, pleckstrin homology domain, SR, spectrin repeat, TBS, Tris Buffered Saline

## Abstract

*TRIO* encodes a cytoskeletal regulatory protein with three catalytic domains—two guanine exchange factor (GEF) domains, GEF1 and GEF2, and a kinase domain—as well as several accessory domains that have not been extensively studied. Function-damaging variants in the *TRIO* gene are known to be enriched in individuals with neurodevelopmental disorders (NDDs). Disease variants in the GEF1 domain or the nine adjacent spectrin repeats (SRs) are enriched in NDDs, suggesting that dysregulated GEF1 activity is linked to these disorders. We provide evidence here that the Trio SRs interact intramolecularly with the GEF1 domain to inhibit its enzymatic activity. We demonstrate that SRs 6-9 decrease GEF1 catalytic activity both *in vitro* and in cells and show that NDD-associated variants in the SR8 and GEF1 domains relieve this autoinhibitory constraint. Our results from chemical cross-linking and bio-layer interferometry indicate that the SRs primarily contact the pleckstrin homology region of the GEF1 domain, reducing GEF1 binding to the small GTPase Rac1. Together, our findings reveal a key regulatory mechanism that is commonly disrupted in multiple NDDs and may offer a new target for therapeutic intervention for *TRIO*-associated NDDs.

The *TRIO* gene encodes a large (>300 kDa) multidomain protein with three catalytic domains (hence the name, Trio): two guanine nucleotide exchange factor (GEF) domains, each composed of Dbl homology (DH) and pleckstrin homology (PH) regions, and a putative serine/threonine kinase domain. The two GEF domains exhibit distinct substrate specificities: the more N-terminal GEF domain (GEF1) promotes GTP loading onto Rac1 and RhoG GTPases (1, 2, 3), while the more C-terminal GEF domain (GEF2) activates RhoA (1, 4, 5). Trio also contains an N-terminal lipid-binding Sec14 domain, nine spectrin repeat (SR) domains, and Src homology 3 and immunoglobulin-like domains (1, 6, 7, 8, 9). Beyond the potential for protein–lipid and protein–protein interactions, the functions of these accessory domains remain poorly understood.

*De novo* mutations and ultra-rare variants in *TRIO* are enriched in neurodevelopmental disorders (NDDs) (10, 11, 12, 13, 14) and the pattern of these variants differs in different disorders. For example, *de novo* missense and rare damaging variants in the GEF1 domain and adjacent regulatory SRs are enriched in autism, intellectual disability, and developmental delay, suggesting that dysregulated GEF1 activity contributes to the pathophysiology of these disorders. Indeed, our lab and others have shown that some of these variants disrupt the ability of GEF1 to catalyze Rac1 activation (12, 13, 14, 15). Clusters of variants in the SR8 and GEF1 domains impacted cellular Rac1 activity in different ways and were associated with distinct endophenotypes in heterozygous carriers: SR8 domain variants were linked to developmental delay, macrocephaly, and hyperactive Rac1 activity in cells, whereas GEF1 domain variants were linked to mild intellectual disability, microcephaly, and reduced Rac1 activity in cells (15). However, the role of the SRs in Trio function and the mechanism of SR8 variant-mediated increase in Rac1 activity are unclear.

Previous studies demonstrated that expression of Trio GEF1 increased Rac1 activity in cells and resulted in dominant gain-of-function pathfinding defects in fly retinal axons (16, 17). Appending additional regions of Trio, including the SRs, to GEF1 attenuated both Trio GEF1-dependent processes. These observations strongly suggest that the SRs reduce GEF1 activity in Trio. However, it remains unknown whether the SRs autoinhibit GEF1 activity directly or *via* the recruitment of cellular cofactor(s). It is also unclear how variants in the SRs would impact this regulatory mechanism *in vitro* and in cells.

We provide evidence here that SRs 6-9 directly inhibit Trio GEF1 activity *in vitro* and in cells. Using a GDP-fluorescein (FL)-BODIPY nucleotide exchange assay (18), we show that inclusion of SRs 6-9 is sufficient to inhibit GEF1 activity *in vitro*, suggesting an autoinhibitory mechanism. We then find that NDD-associated variants in the SR8 and GEF1 domains increase GEF1 activity by relieving autoinhibition, whereas an NDD-associated variant in SR6 reinforces autoinhibition. Using chemical cross-linking and bio-layer interferometry, we demonstrate that the SRs make contact with the PH region of the GEF1 domain and reduce the affinity of GEF1 for Rac1. Together, our findings provide a novel RhoGEF regulatory mechanism by which SRs disrupt Trio GEF1 activation by reducing the interaction of Trio GEF1 with Rac1 and impairing catalytic efficiency. This mechanism appears to be commonly disrupted by NDD-associated variants in *TRIO*, making it a potential target for therapeutic intervention.

## Results

### Inclusion of SRs 6-9 reduces Trio GEF1 activity

**Figure 1:**
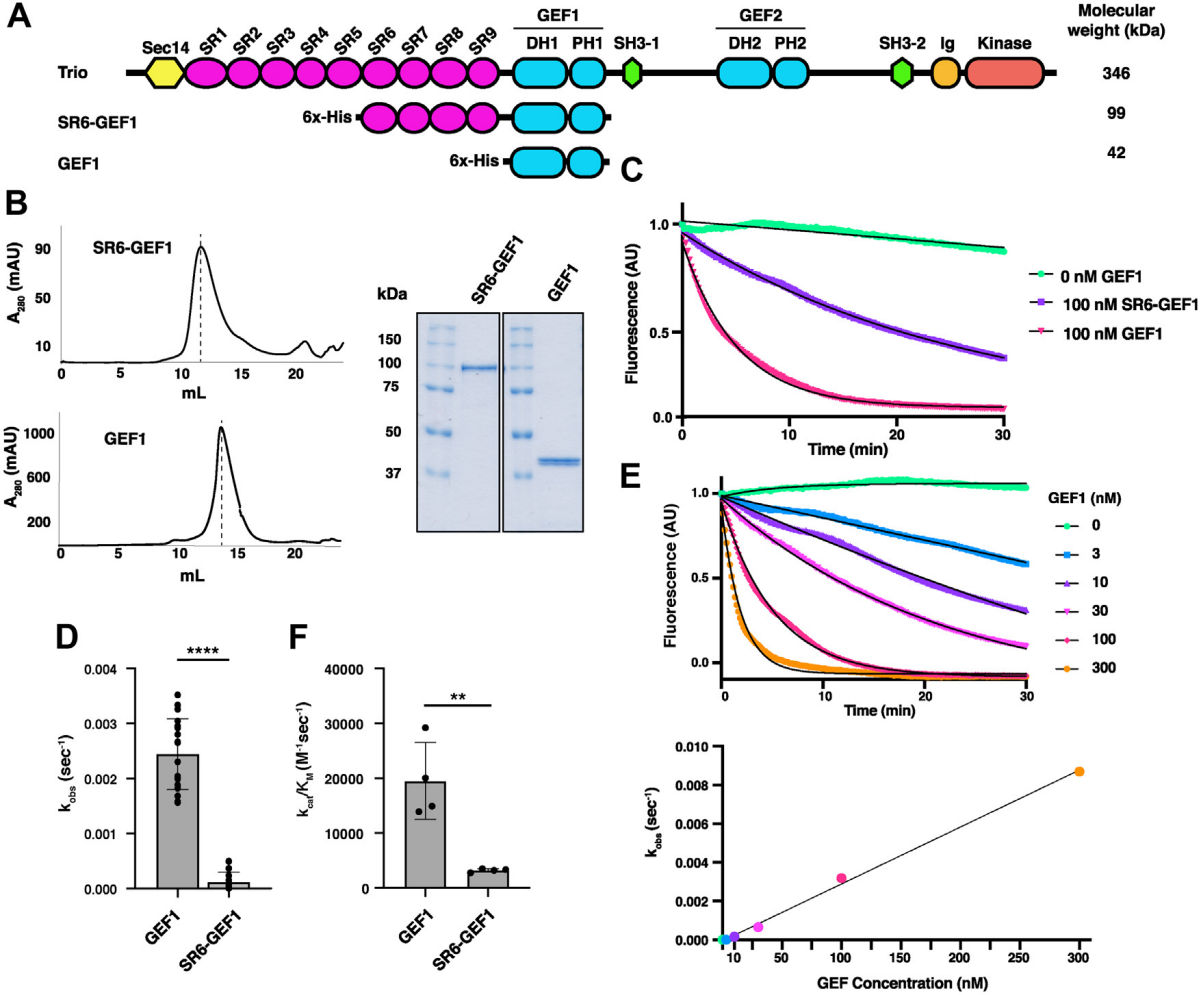
**Inclusion of SRs 6-9 reduces Trio GEF1 activity on Rac1.***A*, schematic of Trio proteins: full-length Trio, SR6-GEF1, and GEF1. *B*, Trio SR6-GEF1 and GEF1 were purified and size-exclusion chromatography was performed to verify that proteins were monodisperse. *Dotted lines* indicate peak elution volume, which is used to calculate Stokes radii. Samples (approximately 5 μg) of purified components were analyzed by SDS-PAGE and stained with Coomassie Blue to assess purity. Gel images were spliced from separate lanes of the same gel, original gel shown in Figure 3*B*. *C*, 100 nM of Trio GEF proteins were incubated with 12.8 μM Rac1 preloaded with 3.2 μM BODIPY-FL-GDP, and nucleotide exchange was tracked *via* the decrease in fluorescence over time. Representative trace is shown here; traces in color, exponential fits overlaid in *black*. *D*, Trio SR6-GEF1 had approximately 20-fold lower exchange activity, k_obs_, than GEF1 alone. N = 21 independent k_obs_ measurements for overall quantification of rates per group. Bars represent average ± SD; ∗∗∗∗*p* ≤ 0.0001 in a two-tailed *t* test. *E*, GEF1 catalytic efficiency was determined by measuring the k_obs_ of GEF1 at multiple concentrations (*top*) and extracting a linear fit from the plot of k_obs_*versus* GEF concentration. Sample traces shown with exponential fits overlaid in *black*. *F*, the catalytic efficiency of SR6-GEF1 was 6-fold lower than GEF1 (n = 4). Bars represent average ± SD of four experimental replicates; ∗∗*p* ≤ 0.005 in a two-tailed *t* test. DH1, Dbl homology domain; FL, fluorescein; GEF, guanine exchange factor; Ig, Ig-like domains; PH1, pleckstrin homology domain; SH3-1, Src homology 3 domain; SR, spectrin repeat.

Genetic variants in SRs 6-9 are associated with NDDs (15), some of which were previously shown to affect Trio-mediated Rac1 activation in cells. To measure the impact of the SRs on GEF1 activity *in vitro*, we generated and purified Trio GEF1 alone (42 kDa) and a Trio fragment containing SRs 6-9 appended to the GEF1 domain (SR6-GEF1, 99 kDa) (Fig. 1*A*). Both proteins were monodisperse upon size-exclusion chromatography and eluted at a position consistent with being monomers (estimated Stokes radius was 3.8 nm for GEF1, 5.6 nm for SR6-GEF1) (Fig. 1*B*). Using a fluorescence-based guanine nucleotide exchange assay, we measured the catalytic activity of GEF1 and SR6-GEF1. Purified 100 nM GEF1 efficiently catalyzed exchange of BODIPY-FL-GDP for GTP on Rac1, with a first-order dissociation rate constant k_obs_ = 2.4 ± 0.6 × 10^−3^ s^−1^ (Fig. 1, C and *D*). Measurement of the rate constant, k_obs_, as a function of GEF1 concentration yielded a k_cat_/K_M_ = 1.9 × 10^4^ M^−1^ s^−1^ (Fig. 1, *E* and *F*). SR6-GEF1 similarly promoted GTP exchange onto Rac1 but with a significantly reduced (∼20 fold and 6-fold, respectively) k_obs_ = 1.2 ± 1.8 ×10^−4^ s^−1^ and k_cat_/K_M_ = 3.1 × 10^3^ M^−1^ s^−1^ (Fig. 1, *C*, *D* and *F*). These data indicate that inclusion of SRs 6-9 inhibits Trio GEF1 activity for Rac1 *in vitro*.

### NDD-associated variants in SR8 increase Trio GEF1 activity in the context of SR6-GEF1

**Figure 2:**
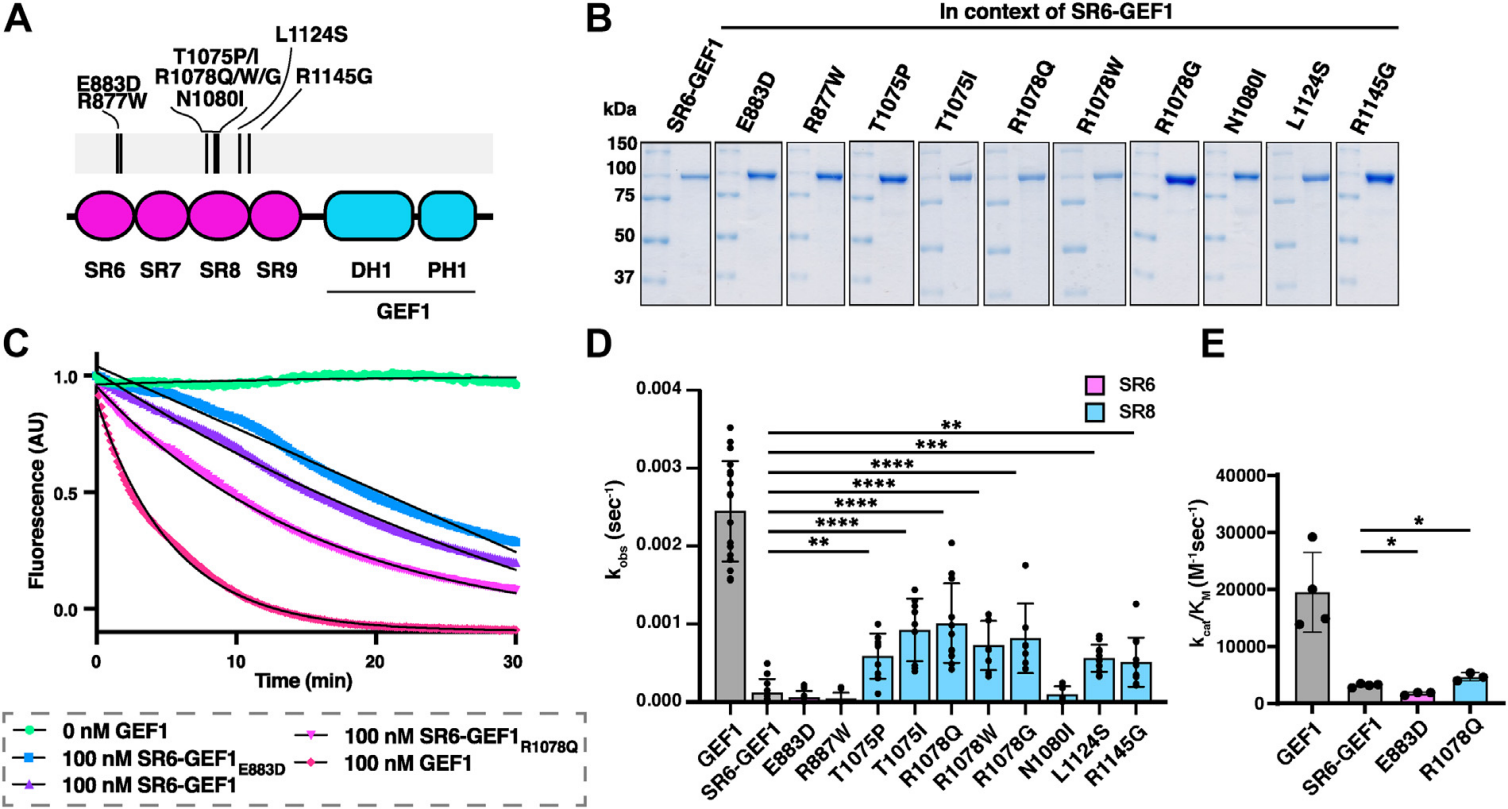
**Mutations in SR6 and SR8 differentially impact GEF1 activity.***A*, schematic of disease associated mutations in the SRs used in this study. *B*, mutants were generated in the context of SR6-GEF1 and purified. *C*, sample GEF assay traces of SR6-GEF1_E883D_ and SR6-GEF1_R1078Q_. Traces in color, exponential fits overlaid in *black*. *D*, SR8 variants in SR6-GEF1 have significantly enhanced catalytic rates, k_obs_, at equal molar amounts (100 nM) (except N1080I). ∗∗*p* ≤ 0.005; ∗∗∗*p* ≤ 0.001; ∗∗∗∗*p* ≤ 0.001 for a significant difference compared to SR6-GEF1 in a one-way ANOVA adjusted for multiple comparisons (n ≥ 9). *E*, catalytic efficiency (k_cat_/K_M_) of representative SR6/8 mutants was determined by measuring the k_obs_ values at different concentrations of GEF, as shown in Figure 1*D*. The catalytic efficiency of SR6-GEF1_R1078Q_ is ∼1.5-fold greater than that of SR6-GEF1, while the catalytic efficiency of SR6-GEF1_E883D_ is ∼1.8-fold slower (n = 3). Data for GEF1 and SR6-GEF1 from Figure 1 are shown again for reference, and all are reported as an average ± SD of three or more experimental replicates. ∗ = significantly different from SR6-GEF1, *p* ≤ 0.05 in a one-way ANOVA adjusted for multiple comparisons. GEF, guanine exchange factor; SR, spectrin repeat.

We generated and purified SR6-GEF1 expression constructs containing single NDD-associated variants in SR8 and measured their ability to catalyze nucleotide exchange on Rac1 (Fig. 2, *A* and *B*). When tested at 100 nM, all SR8 variants, except N1080I, increased the k_obs_ by 4 to 8 fold over that of WT SR6-GEF1 (Fig. 2, *C* and *D*). In agreement with these findings, one representative SR8 variant, SR6-GEF1_R1078Q_, which had a significantly increased k_obs_ = 1.0 ± 0.5 × 10^−3^ s^−1^, had a k_cat_/K_M_ = 4.7 × 10^3^ M^−1^ s^−1^, a 1.5-fold increase in catalytic efficiency over WT SR6-GEF1 (Fig. 2*E*). These findings indicate that NDD-associated variants in SR8 are sufficient to relieve SR autoinhibition.

### NDD-associated variants in SR6 decrease GEF1 activity in the context of SR6-GEF1

We also generated two SR6-GEF1 constructs harboring individual disease variants in the SR6 domain. While the rate constant (k_obs_) values obtained for each construct did not significantly decrease compared to WT SR6-GEF1, measurement of catalytic efficiency, k_cat_/K_M_, of both WT SR6-GEF1 and SR6-GEF1_E883D_ revealed that SR6-GEF1_E883D_ had a significantly decreased catalytic efficiency of a k_cat_/K_M_ = 1.7 × 10^3^ M^−1^ s^−1^, 1.8-fold lower than WT SR6-GEF1 (Fig. 2*E*). This suggests that NDD-associated variants in SR6 decrease GEF1 activity.

### GEF1 variant D1368V increases GEF activity only in the context of SR6-GEF1

**Figure 3:**
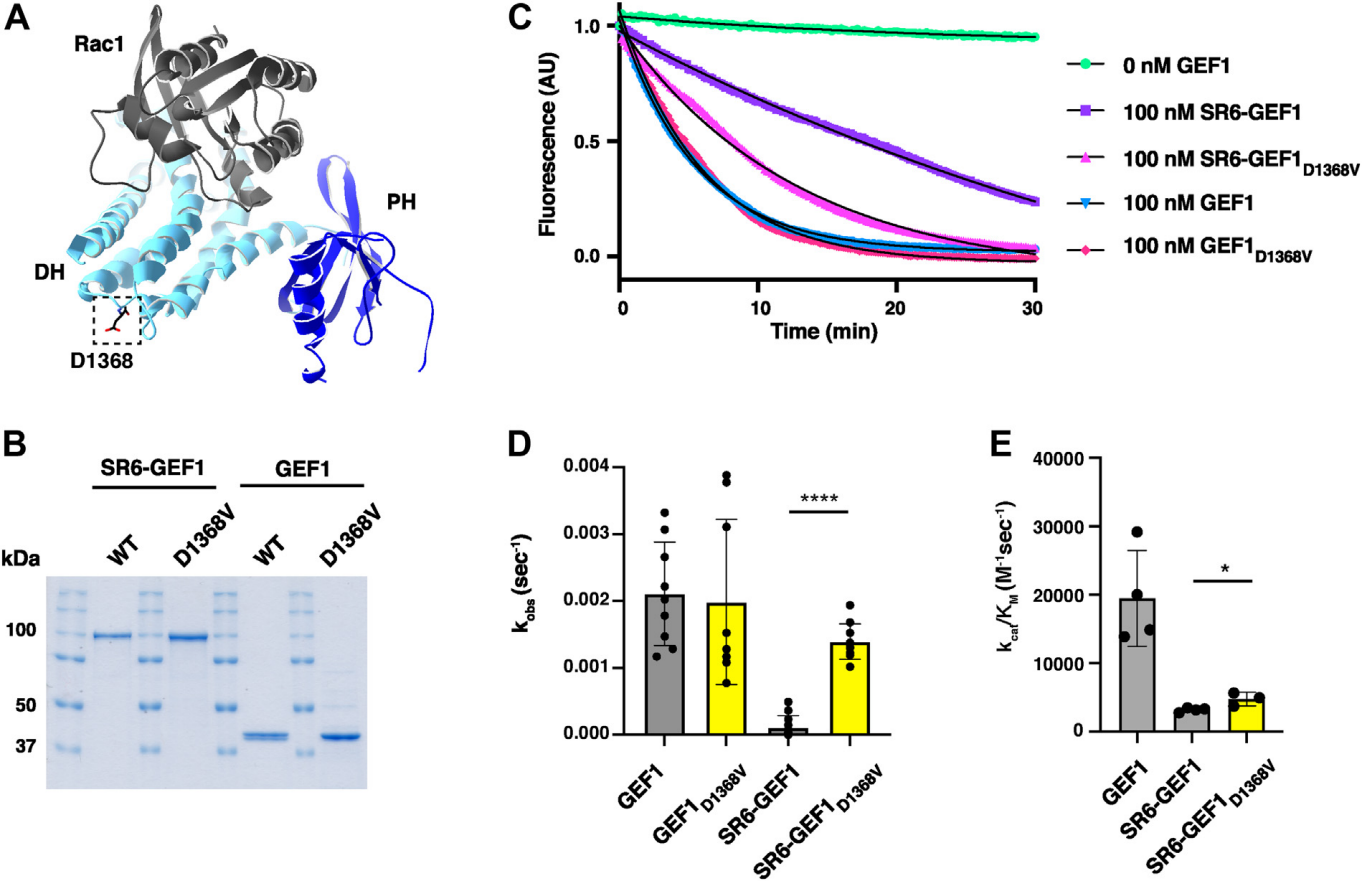
**GEF1 variant D1368V increases GEF1 activity in the context of SR6-GEF1.***A*, crystal structure of Trio GEF1 (*light* and *dark blue*) and Rac1 (*gray*), accessed in PDB, ID = 2NZ8 (5). D1368, identified in the *box*, is distal to the Rac1-binding interface. *B*, samples (approximately 5 μg) of purified components were analyzed by SDS-PAGE and stained with Coomassie Blue R250 to assess purity. Gel bands for WT SR6-GEF1 and WT GEF1 are the same as shown spliced in Figure 1*B*. *C*, sample GEF assay traces of D1368V in the context of SR6-GEF1 and GEF1. Traces in color, exponential fits overlaid in *black*. *D*, D1368V in SR6-GEF1 increases catalytic rate, k_obs_, at equal molar amounts of GEF but has no impact when inserted into GEF1 alone (∗∗∗∗*p* ≤ 0.0001, unpaired *t* test for mutant *versus* WT in respective GEF1 or SR6-GEF1, n = 3). *E*, catalytic efficiency (k_cat_/K_M_) of SR6-GEF1_D1368V_ was determined by measuring the k_obs_ values at different concentrations of GEF, as in Figure 1*D*. Data for GEF1 and SR6-GEF1 shown again for reference. The catalytic efficiency, k_cat_/K_M_, of SR6-GEF1_D1368V_ is ∼1.5-fold greater than that of SR6-GEF1 (n = 3). ∗ = significantly different from SR6-GEF1, *p* ≤ 0.05 in a one-way ANOVA adjusted for multiple comparisons. GEF, guanine exchange factor; SR, spectrin repeat.

Hypothesizing that the SRs might contact GEF1 to impact catalytic activity, we searched for GEF1 domain variants that might impact potential autoinhibition of GEF1 activity by SRs. Unlike GEF1 disease variants that lie in the GEF1:Rac1 interface and decrease GEF1 activity (12, 13, 14), D1368V lies in the DH domain but is distal to the GEF1:Rac1 interface, so its impact is less well understood (Fig. 3*A*). However, introduction of the D1368V variant greatly potentiates the ability of the Trio9 splice isoform, which contains all of the SRs, to increase activity of a Rac1 reporter in cells (14). We introduced D1368V into SR6-GEF1 and found that it significantly increased catalytic activity, with a k_obs_ = 1.4 ± 0.3 × 10^−3^ s^−1^ and k_cat_/K_M_ = 4.8 × 10^3^ M^−1^ s^−1^ (Fig. 3, *B*–*E*), a 1.5-fold increase over the k_cat_/K_M_ for WT SR6-GEF1. In contrast, introducing D1368V into GEF1 alone did not impact its activity compared to GEF1 (Fig. 3, *B*–*E*), indicating that the activating effects of D1368V require SRs 6-9. Together with data reported above, these are consistent with a model in which NDD-associated variants in SR8 and GEF1 relieve inhibition of GEF1 activity by the SRs.

### The SRs and GEF1 form distinct stable interacting domains

**Figure 4:**
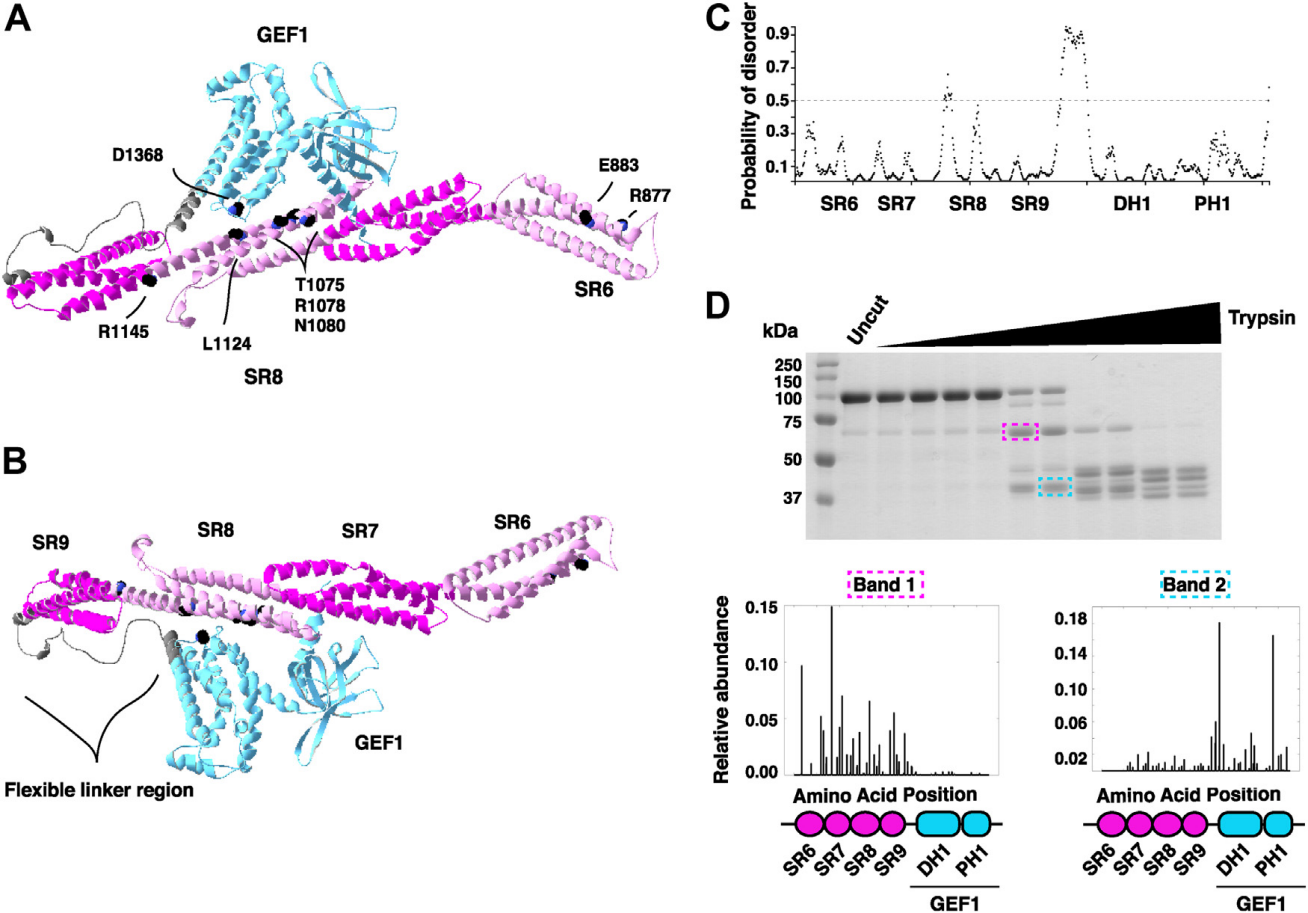
**AlphaFold predicts an interaction between the SRs and GEF1, which form independent folding units.***A*, AlphaFold model of human Trio SR6-GEF1. SR6, 8 in *light pink*, SR7, 9 in *dark pink*, linker region in *gray*, and GEF1 in *blue*. Sites of mutations used in this study are modeled as *black spheres*, with amino acids labeled. This model predicts an interaction between SR8 and GEF1. *B*, SR6-GEF1 from AlphaFold model, rotated to view flexible linker region between GEF1 and SR9. *C*, probability of disorder was predicted using DISOPRED. The region between SR9 and DH1 has a high probability of being disordered (cutoff > 0.5). *D*, limited proteolysis of SR6-GEF1. His-SR6-GEF1 was incubated with increasing concentrations of trypsin and select bands were identified using mass spectrometry. Relative abundance of identified peptides was plotted to determine composition of each band. The y-axis displays relative abundance of peptides and x-axis is ‘amino acid position’, which refers to the location in SR6-GEF1 that the peptide covers (with SR6-GEF1 diagram below). Band 1 (*pink box* around gel band at ∼60 kDa) comprises SR6-9 and Band 2 (*blue box* around band at ∼40 kDa) comprises GEF1. Therefore, SR6-9 and GEF1 form distinct stable domains. DH1, Dbl homology domain; GEF, guanine exchange factor; SR, spectrin repeat.

We used AlphaFold (19, 20) to model human Trio SR6-GEF1 (Fig. 4, *A* and *B*). Strikingly, this model suggests that SRs interact with the GEF1 domain, with SR8 closely apposed to GEF1 and the NDD-associated mutations concentrated at this SR8:GEF1 interface. This model of SR6-GEF1 and additional analysis using DISOPRED predicted the existence of an unstructured loop between SR9 and GEF1, suggesting this flexible region may connect the SRs and GEF1 domain (Fig. 4*C*) (21). We used limited proteolysis to probe for the presence of a flexible linker between SR9 and the GEF1 domain that might be susceptible to partial proteolysis. Treatment of SR6-GEF1 at intermediate levels of trypsin yielded two major bands, identified by mass spectrometry as composed of SRs 6-9 and GEF1, respectively. This observation indicates that SRs 6-9 and the GEF1 domain each make up distinct folding units with increased relative resistance to protease (Fig. 4*D*). Together, these findings support a model in which the SRs make contact with GEF1.

**Figure 5:**
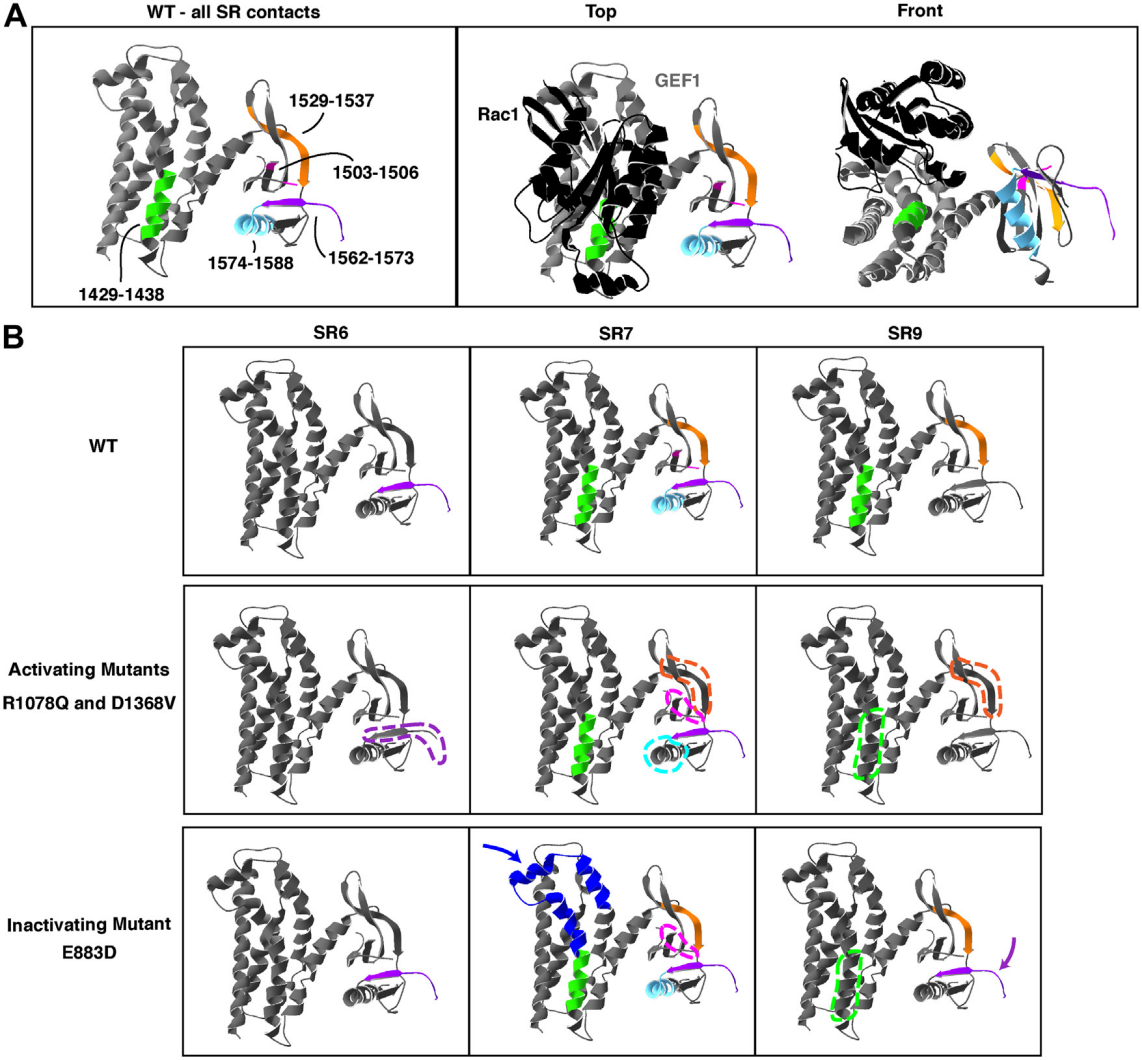
**The SRs interact with GEF1.***A*, SR6-GEF1 was incubated with lysine cross-linker BS3 and cross-linked peptides were identified using mass spectrometry. Crystal structure of GEF1 alone (*gray*, *left panel*) and with Rac1 (*black*, *right panel*) (from PDB, ID = 2NZ8 (5)) with cross-linked peptides between SR6-9 and GEF1 (in WT case) shown in *green* (1429–1438), *pink* (1503–1506), *orange* (1529–1537), *purple* (1562–1588), and *light blue* (1574–1588). SR6-9 contacts the DH domain at a peptide that likely interferes with Rac1 binding (1429–1438) and a region in the PH domain critical for stabilizing the Rac1 interaction (1529–1537) (5). *B*, representative activating mutants (R1078Q and D1368V) display fewer contacts between SR6-9 and GEF1 (lost contacts shown with *dotted lines*). Representative inactivating mutant (E883D) displays increased contacts between SR6-9 and GEF1 (New contacts shown with *blue* or *purple arrows*). Cross-links were categorized based on their N-terminal cross-link site (in SR6, 7, or 9) and their C-terminal GEF1 contacts were visualized. For the activating mutants, the peptides that were mutually lost for both activating mutants were visualized here. For table of all mutant cross-links between SR6-9 and GEF1, see Table S2. BS3, bis(sulfosuccinimidyl)suberate; DH1, Dbl homology domain; GEF, guanine exchange factor; PH1, pleckstrin homology domain; SR, spectrin repeat.

To test directly for possible interactions between the SRs and GEF1 domain, we incubated SR6-GEF1 with an 11.4 Å spacer lysine cross-linker, BS3 (bis(sulfosuccinimidyl)suberate), and analyzed cross-linked peptides *via* mass spectrometry to identify sites in close enough proximity to cross-link. Several long-distance cross-links were observed between the SRs and the GEF1 domain (Fig. 5*A*). Specifically, the SR:GEF1 interface includes a peptide in DH domain which is directly at the Rac1 binding interface (1429–1438, green in Fig. 5*A*) and a peptide in the PH domain important for stabilizing the Rac1 interaction (1529–1537, orange in Fig. 5*A*) (Fig. 5*A*) (5). Multiple regions originating in SR6-9 contact these peptides in the GEF domain. This suggests that SR6-GEF1 may be dynamic, with multiple conformational states captured by cross-linking. We hypothesize that these SR:GEF1 contacts likely disrupt Rac1 binding to GEF1

We also performed chemical cross-linking on three variants in SR6-GEF1 to understand how intramolecular contacts may change in the variants. The SR6-GEF1 variants that display activated GEF activity, R1078Q and D1368V, both exhibited a loss of contact between SR6, 7, 9, and the GEF1 domain (Fig. 5*B*). In addition, R1078Q, but not D1368V, also reduced SR8:GEF1 contacts (Table S2). In contrast, the SR6 variant, E883D, which reduced GEF activity, did not reduce intramolecular contacts with GEF1; in fact, new contacts appeared (SR7 and SR9 contacts, blue and purple arrowheads, Fig. 5*B*), suggesting this variant may reinforce intramolecular SR:GEF contacts (Fig. 5*B*). These data are consistent with a model in which specific intramolecular contacts between the SRs and GEF1 are altered in genetic variants with increased GEF1 activity.

### The SRs reduce GEF1 binding to Rac1

**Figure 6:**
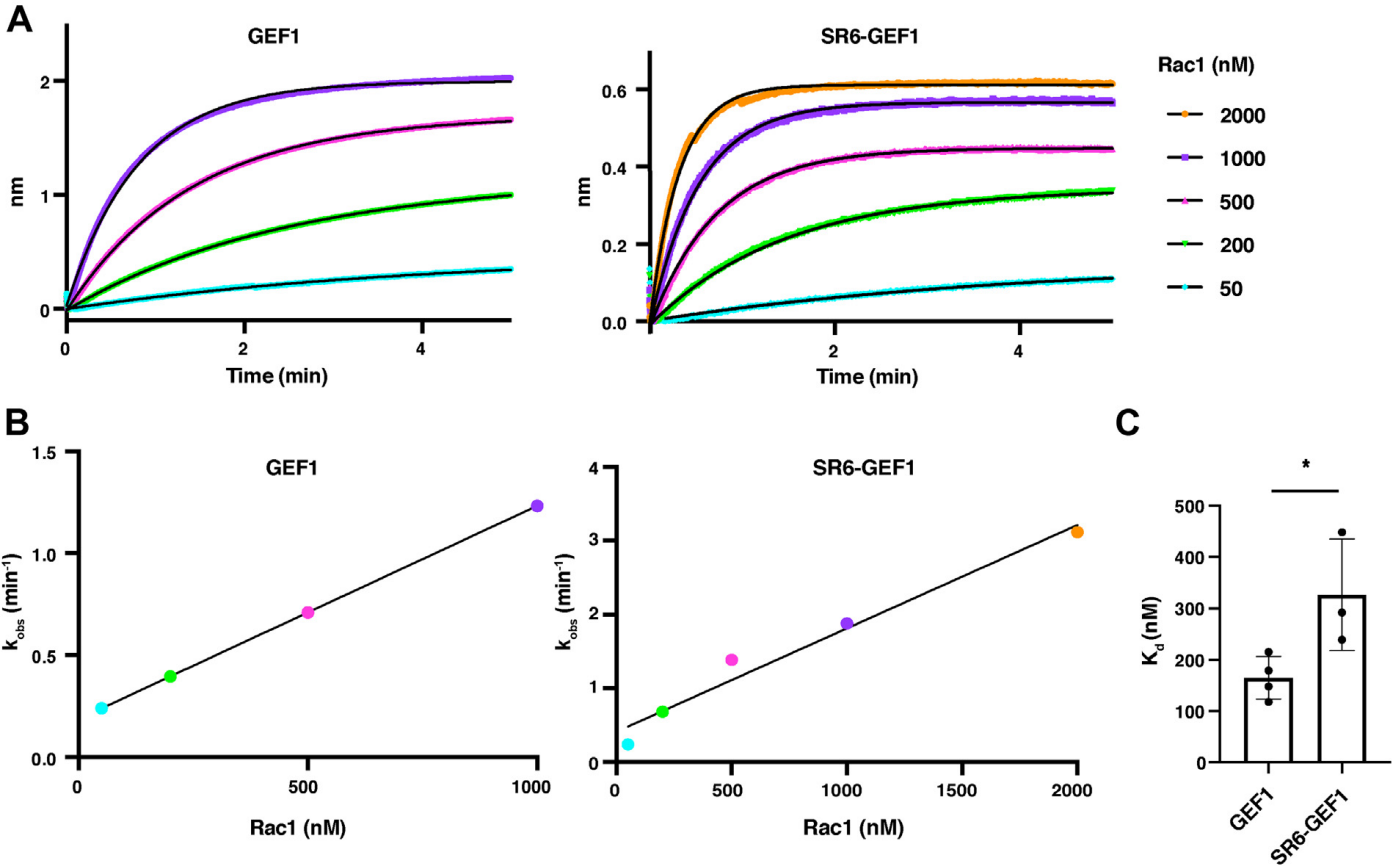
**Inclusion of SRs 6-9 reduce binding to Rac1.***A*, His-GEF1 or His-SR6-GEF1 were immobilized on an Ni-NTA biosensor and the association of different concentrations of Rac1 was measured. Representative traces shown, with data in color and one phase exponential fits in *black*. Full concentration gradients (4–5 Rac1 concentrations) were performed at least three independent times. *B*, k_obs_ values were extracted from each association curve and plotted against Rac1 concentration to calculate a K_d_ of GEF1 or SR6-GEF1 binding to Rac1. *C*, SR6-GEF1 has a 2-fold weaker affinity for Rac1 than GEF1 (∗*p* ≤ 0.05, unpaired *t* test). GEF, guanine exchange factor; Ni-NTA, nitrilotriacetic acid; SR, spectrin repeat.

Based on our cross-linking data, we hypothesized that an interaction between SRs 6-9 and PH1 may impair the ability of GEF1 to bind Rac1. We used bio-layer interferometry to measure the association of nucleotide-free Rac1 with His-GEF1 or His-SR6-GEF1 immobilized on a nitrilotriacetic acid (Ni-NTA) affinity chip. GEF1 bound to Rac1 with a K_d_ = 151 ± 49 nM in nucleotide-free conditions (Fig. 6, *A*–*C*). SR6-GEF1 had a reduced affinity for Rac1, with a K_d_ = 316 ± 87 nM (Fig. 6, *A*–*C*). Taken together with the cross-linking data, this supports a model where the SRs contact the PH domain to impair GEF1 binding to Rac1, which likely contributes to the reduction in observed GEF1 activity.

### SRs 6-9 inhibit GEF1-induced cell spreading

**Figure 7:**
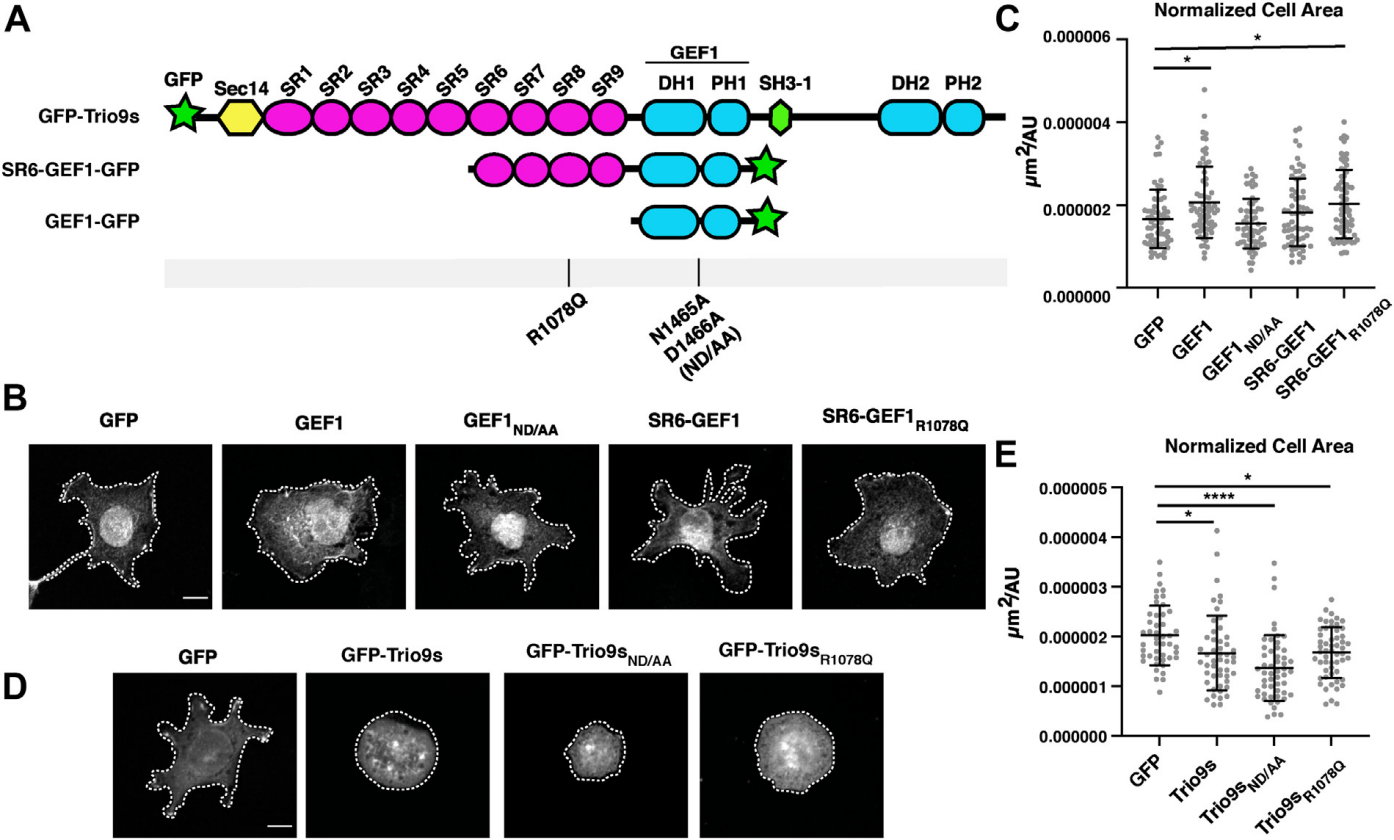
**SRs 6-9 reduce the impact of GEF1 on cell spreading.***A*, schematic of constructs used, with mutants shown below. *B*, constructs in (*A*) were transfected into HEK293 cells and plated on fibronectin. Cells were fixed and stained using anti-GFP to visualize GFP expression and cell morphology. Cells expressing GEF1 and SR6-GEF1_R1078Q_ appeared to have more rounded edges and circular shapes. The scale bar represents 10 μm. Contrast was adjusted between images shown to best visualize cell edge; cell edge is outlined with a *white dashed line*. *C*, cell area, normalized to protein expression on a cell-by-cell basis, was quantified. Cell area increased upon expression of GEF1 and SR6-GEF1_R1078Q_, while expression of a catalytic-dead GEF1 mutant (ND/AA) or SR6-GEF1 had no effect compared to GFP alone. *D*, cells visualized and analyzed as in (*B*). The scale bar represents 10 μm. Cells expressing Trio9s constructs all appeared rounder and lacked cell edge protrusions. *E*, cell area quantified as in (*C*). Cell area was decreased upon expression of all GFP-Trio9s constructs compared to GFP alone. Trio9s_R1078Q_ did not increase cell area to levels seen with GFP alone. Two biological replicates were performed for each set of constructs, with 25 to 40 cells analyzed per group per replicate (∗*p* ≤ 0.05, ∗∗∗∗*p* ≤ 0.0001, one-way ANOVA between GFP control and each group and adjusted for multiple comparisons). GEF, guanine exchange factor; SR, spectrin repeat.

Trio GEF1 activates Rac1 and RhoG to coordinate downstream cytoskeletal changes and mediate changes in cell morphology (1, 2, 3, 22). We first expressed Trio GEF1-GFP in HEK293 cells and quantified its impact on cell morphology (Fig. 7, *A*–*C*). When matched for GFP expression levels, GEF1 expressing cells had significantly increased cell area compared to GFP controls (Fig. 7, *A*–*C*). Cells expressing GEF1 appeared to be more spread with round lamellipodia encompassing the cell edge, a common result of Rac1 activation (23) (Fig. 7*B*). The area of cells expressing a catalytic-dead mutant of GEF1, GEF1 ND/AA (N1465A/D1466A), were similar to GFP controls, indicating a key role for GEF1 catalytic activity in this morphological change (24). In contrast to GEF1, SR6-GEF1 expressing cells had no measurable effect on cell area, but the SR8 mutant, SR6-GEF1_R1078Q_, increased cell area over that of GFP and SR6-GEF1 WT (Fig. 7, *B* and *C*). Cells expressing SR6-GEF1_R1078Q_ also appeared qualitatively similar in morphology to those cells expressing GEF1 alone, with more full, rounded edges (Fig. 7*B*). Therefore, inclusion of SRs 6-9 inhibits Trio GEF1-dependent changes in cell morphology, and disease-associated variants can disrupt this inhibitory regulation.

We then expressed GFP-Trio9s, a predominant neuronal isoform throughout neurodevelopment, in HEK293 cells and quantified its impact on cell morphology (25) (Fig. 7, *A*, *D* and *E*). Interestingly, when matched for GFP expression levels, GFP-Trio9s expressing cells had significantly decreased cell area compared to GFP controls. Expressing two variants of Trio9s, the most activated SR8 mutant, GFP-Trio9s_R1078Q_, and a catalytic-dead mutant of GEF1, GFP-Trio9s ND/AA (N1465A/D1466A), decreased cell area compared to GFP alone (Fig. 7, *D* and *E*). Cells expressing any variant of GFP-Trio9s appeared very round, completely lacking lamellipodia or cell edge protrusions (Fig. 7, *D* and *E*). We speculate that activity of the Trio GEF2 domain, which targets RhoA to promote cytoskeleton contractility (26), may dominate in this context, making it difficult to discern specific effects on GEF1 activity.

## Discussion

We provide evidence here that the Trio SRs 6-9 directly inhibit GEF1 activity *via* intramolecular interactions *in vitro* and in cells. We demonstrate that NDD-associated variants in the SR8 and GEF1 domains release this autoinhibitory constraint, strongly suggesting that disruption of this GEF1 regulatory mechanism contributes to the pathophysiology of these disorders. Using chemical cross-linking and bio-layer interferometry, we show that the SRs contact regions of GEF1 important for Rac1 binding and that inclusion of the SRs is associated with reduced binding affinity for Rac1 *in vitro*. We present a model for how Trio GEF1 activity is regulated, and how this regulation is disrupted by disorder-associated variants.

### Inclusion of Trio SRs autoinhibits GEF1 activity *in vitro*

Previous cell-based studies have shown that removing the SRs is associated with increased downstream Rac1 activity and Trio gain-of-function phenotypes *in vivo*, suggesting that the Trio SRs function to inhibit GEF1 activity (16, 17, 27). This hypothesis is supported by evidence that other RhoGEFs, like Tiam1, contain autoinhibitory N-terminally adjacent accessory domains (8, 28, 29). In most cases, how inhibition occurs and how it is released to activate GEF activity is unknown. Our results show that SR6-GEF1 is monomeric in solution and that inclusion of SRs significantly decreases GEF1 catalytic activity *in vitro*. Collectively, these observations suggest that the SRs are sufficient to inhibit GEF1 activity *via* intramolecular interactions in *cis*.

### SRs make direct contact with GEF1 and impair interactions with Rac1

Within GEF1, the DH1 domain catalyzes GTP exchange onto Rac1 and serves as the main Rac1-binding interface. The PH domain plays a regulatory role in catalysis but also serves to stabilize the Rac1:DH1 interaction (30, 31). Using chemical cross-linking, we demonstrate that SRs 6-9 make extensive contacts with the GEF1 domain, including at sites critical for Rac1 binding, suggesting that SR6-9 *sterically* blocks contact with Rac1. In addition, NDD-associated variants that activate GEF1 exhibit reduced contacts between the SRs and GEF1 and those that impair GEF1 activity exhibit increased contacts. Hence, altering the interaction between the SRs and GEF1 impacts catalytic activity (5).

We found that inclusion of SRs 6-9 reduces the affinity of GEF1 for Rac1 by 2-fold, compared to GEF1 alone. Whereas our catalytic rate measurements suggest the presence of SRs 6-9 results in a 6-fold decrease in activity, the reduction in affinity that we observed was smaller in magnitude. It is likely that engagement of the SRs with GEF1 impairs other steps in the catalytic cycle, as demonstrated by our catalytic efficiency data, in addition to impacting Rac1-binding affinity. Future studies will elucidate whether other components of the nucleotide exchange process are impacted by the SRs.

### NDD-associated mutations in SR8 and GEF1 disrupt SR-mediated GEF1 inhibition

Two rare variant clusters in *TRIO*, one in SR8 (Fig. 2*A*) and one in GEF1, have been linked to distinct endophenotypes in individuals with NDDs (15). For example, *TRIO* SR8 variants are linked to developmental delay and macrocephaly in humans and cause increased Rac1 (GEF1) activity in cells, whereas most mutations in the GEF1 domain are linked to mild intellectual disability, microcephaly, and reduced Rac1 activity in cells. However, how SR8 variants increased Rac1 activity was completely unknown. We hypothesized that the increased Rac1 activity associated with SR8 domain variants resulted from disruption of SR-mediated GEF1 inhibition. We generated mutant SR6-GEF1 constructs harboring distinct disorder-associated variants and found that nearly all SR8 mutants increased SR6-GEF1 catalytic activity 4 to 8 fold. Interestingly, the one exception, N1080I, disrupts binding to neuroligin-1 and blocks neuroligin-1–mediated synaptogenesis (32). We hypothesize that other sites, including N1080I, in the SRs serve as convergence points for upstream activators to regulate GEF1 activity and discuss this in a following section. Together, these data demonstrate that many NDD variants in SR8 are sufficient to relieve SR-mediated GEF1 inhibition.

We also found that a GEF1 domain variant associated with Rac1 activation in cells likely impacts SR-mediated GEF1 inhibition. Unlike GEF1 disease variants that lie at the Rac1-binding interface and decrease GEF1 activity, this variant, D1368V, is distal to the Rac1 interface and hyperactivates Rac1 activity in cells when introduced in the Trio9 splice isoform (12, 13, 14, 32). Our results indicate that D1368V significantly increases GEF1 activity in the context of SR6-GEF1 but has no effect on GEF1 alone. We propose that D1368V enhances SR6-GEF1 activity by disrupting SR autoinhibition. Indeed, our cross-linking data suggests that contacts between the SRs and GEF1 are reduced for the D1368V variant.

### NDD-associated variants in SR6 may reinforce SR-mediated GEF1 inhibition

We also generated two SR6-GEF1 constructs harboring individual disease variants in the SR6 domain, whose impact on Trio function remains completely unknown. The catalytic efficiency (k_cat_/K_M_) of SR6-GEF1_E883D_ was significantly slower than SR6-GEF1, suggesting that SR6 mutants decrease SR6-GEF1 catalytic activity. While the mechanism for this is unclear, one possibility is that SR6 acts as a hinge region allosterically governing the flexibility of the helices surrounding SR8 and that SR6 variants may decrease the ability for the SRs to release their inhibitory lock on the GEF1 domain. Indeed, we observed more contacts between SR7 and SR9 and the GEF1 domain in SR6-GEF1_E883D_, suggesting that the intramolecular contacts are more stable or extensive in the variant case. This observation underscores the importance of understanding how dysregulation of Trio GEF1 activity contributes to NDDs.

### The SRs may serve as a target for activators of Trio GEF1 activity

We demonstrated that the SRs inhibit Trio GEF1 activity, but it is unclear how inhibition may be released in a cellular context. SR domains are widely accepted as scaffolding proteins that coordinate cytoskeletal interactions with high spatial precision. Considering that Trio is known to act downstream of cell surface receptors to coordinate cytoskeletal rearrangements, we anticipate that the Trio SRs serve as a target of interaction partners to engage and activate Trio GEF1 activity in cells. Trio SRs interact with diverse cellular partners, including synaptic scaffolding proteins (Piccolo and Bassoon) (33), cell-adhesion molecules (VE-cadherin and Intercellular Adhesion Molecule 1 (ICAM1)) (34, 35), and membrane trafficking proteins (RABIN8) (36). These SR-binding partners may engage Trio to coordinate GEF1 activation and/or deactivation in a spatiotemporal manner. Indeed, several studies have shown that Trio interactions with binding partners impacts Rac1 activity in cells (32, 34, 35, 37, 38). For example, VE-cadherin binds Trio SR5 and SR6, and this interaction locally increases Rac1 activity in cells (34). Similarly, the ICAM1 intracellular tail binds Trio GEF1, and the Trio/ICAM1 interaction potentiates ICAM1 clustering at adhesion sites, promoting Rac1 activation in cells (35). Finally, the integral membrane protein Kidins220 regulates Rac1-dependent neurite outgrowth *via* interactions with the Trio SRs (37). While these studies suggest that the Trio signaling partners may engage and activate Trio GEF1 activity, the specific interaction interfaces and binding stoichiometry that mediates GEF1 activation and how they are impacted by disorder-associated variants is presently unknown. Based on our evidence that SR8 variants relieve autoinhibitory constraint, we anticipate that SR8 may be a convergence point for upstream activators and coordinated regulation of GEF1 activity.

## Conclusions

*TRIO* has emerged as a significant risk gene for NDDs. Using biochemical and genetic tools, we identified a novel regulatory mechanism by which Trio SRs inhibit GEF1 activity and showed that disorder-associated variants are sufficient to relieve this autoinhibitory constraint. This discovery will serve as a model to understand how Trio GEF1 is regulated by physiological signals and how its disruption leads to NDDs. This mechanism may also offer a new target for therapeutic interventions for *TRIO*-associated NDDs.

## Experimental procedures

### Expression construct cloning and protein purification

Human Trio SR6-GEF1 was PCR amplified and inserted into the pFastBac1 HTa vector (Invitrogen). Site-directed mutagenesis was used to insert point mutations into pFastBac1-Hta-SR6-GEF1 construct and confirmed by DNA sequencing. Primers used for cloning are included in Table S1.

Recombinant baculoviruses were generated using Sf9 cells (Bac-to-Bac expression system, Thermo Fisher Scientific). Baculoviruses were used to infect Hi5 cells at an estimated multiplicity of infection = 1 for 48 h before lysis in lysis buffer (20 mM Hepes pH 7.25, 500 mM KCl, 5 mM *β*-mercaptoethanol, 5% glycerol, 1% TritonX-100, 20 mM imidazole, 1 mM DTT, 1 mM PMSF, 1× Roche cOmplete protease inhibitors EDTA free) for 20 min at 4 °C. Lysates were affinity purified using Ni-NTA resin (Qiagen) and eluted with 250 mM imidazole. Elution fractions were further purified over an Sephadex 200 (S200) Increase 10/300 GL column into assay buffer (20 mM Hepes pH 7.25, 150 mM KCl, 5% glycerol, 0.01% TritonX-100, 1 mM DTT), aliquoted, and flash frozen for long-term storage.

Human Trio GEF1 and Rac1 were generated and affinity purified from bacterial cells as described in Blaise *et al.* (18). Point mutants were generated using site-directed mutagenesis. Following affinity purification, eluted protein was further purified over an S200 Increase column into assay buffer, aliquoted, and flash frozen for long-term storage.

Stokes radii of proteins were estimated based on the elution volume from the S200 Increase column, calculated based on a standard curve generated by running protein standards (Protein Standard Mix 15–600 kDa, Supelco).

### BODIPY-FL-GDP nucleotide exchange assays

12.8 μM Rac1 was loaded with 3.2 μM BODIPY-FL-GDP (Invitrogen) in 1× assay buffer (20 mM Hepes pH 7.25, 150 mM KCl, 5% glycerol, 1 mM DTT, 0.01% TritonX-100) plus 2 mM EDTA to a total volume of 25 μl per reaction, then incubated for 1 h at room temperature. BODIPY-FL-GDP loading onto Rac1 was halted by the addition of 5 μl of MgCl_2_, for a total reaction volume of 30 μl with a final MgCl_2_ concentration of 5 mM. Prior to initiating the reaction with 100 nM Trio GEF, 30 μl of GTPase (12.8 μM) plus MgCl_2_ (5 mM) mix or blank (3.2 μM BODIPY-FL-GDP, 2 mM EDTA, and 1× assay buffer) was added to appropriate wells. During the BODIPY-FL-GDP loading incubation period, GEF1-containing proteins were prepared in 1× assay buffer, 4 mM GTP, and 2 mM MgCl_2_. Exchange reactions were initiated by adding 10 μl of 100 nM Trio GEF mixture (as stated above) to each well, for a total reaction volume of 40 μl. Real-time fluorescence data was measured every 10 s for 30 min monitoring BODIPY-FL fluorescence by excitation at 488 nm and emission at 535 nm, as per Blaise *et al.* (18).

All k_obs_ measurements of GEF1 activity represent at least three experimental replicates with three technical replicates per experiment. Results are shown as the mean ± SD from multiple experiments. A one-way ANOVA was used to determine statistical significance between SR6-GEF1 and all other variants (two-tailed *p*-value < 0.05) and adjusted using Dunnett’s multiple comparisons test. Catalytic efficiencies (k_cat_/K_M_) of selected SR6-GEF1 constructs were extracted from a linear fit of catalytic rate (k_obs_, s^−1^) *versus* GEF1 concentration (nM). Three experimental replicates were performed for each SR6-GEF1 construct, and the catalytic efficiency values were averaged. Results are shown as the mean ± SD. A one-way ANOVA was used to determine statistical significance between SR6-GEF1 and all other variants (two-tailed *p*-value < 0.05) and adjusted using Dunnett’s multiple comparisons test.

### Protein structure predictions

AlphaFold was used to access the predicted structure of human Trio spectrin repeats 1-GEF1 (amino acids 201–1600), entry number AF-O75962-F2 (19, 20). Swiss pdb Viewer was used to model SR6-GEF1, amino acids 788 to 1599 (39). DISOPRED was used to predict the probability of disorder of Trio SR6-GEF1, amino acids 788 to 1599 (21).

### Limited proteolysis

SR6-GEF1 in assay buffer plus 10 mM CaCl_2_ was diluted to 0.4 mg/ml and incubated with increasing concentrations of trypsin (0.001 mg/ml–0.11 mg/ml) for 1 h at room temperature in a 25 μl total reaction volume. Reactions were quenched with 8 μl quench buffer (50 mM Tris–HCl pH 6.8, 4% SDS, 10% glycerol, 0.1% bromophenol blue, 5% *β*-mercaptoethanol, 1 mM PMSF, 4 mM EGTA, 4 mM EDTA) and immediately boiled for 10 min. Samples were immediately run on a 12% SDS-PAGE gel, and proteins were visualized by Coomassie R250 staining.

Major gel bands were excised and washed with 50:50 acetonitrile:water buffer containing 100 mM ammonium bicarbonate. Proteins in the gel were reduced with 4.5 mM DTT at 37 °C for 20 min and alkylated with 10 mM iodoacetamide at room temperature for 20 min in the dark. Gel bands were washed twice with 50:50 acetonitrile:water containing 100 mM bicarbonate and dried for 10 min in a SpeedVac. Trypsin digestion was carried out (1:100 M ratio of trypsin to protein) by incubation with the gel piece at 37 °C overnight. The digest samples were analyzed by LC–MS/MS using a Q-Exactive Plus mass spectrometer equipped with a Waters nanoACQUITY ultra-performance liquid chromatography system using a Waters Symmetry C18 180 μm by 20 mm trap column and a 1.7 μm (75 μm inner diameter by 250 mm) nanoACQUITY ultra-performance liquid chromatography column (35 °C) for peptide separation. Trapping was done at 15 μl/min with 99% buffer A (100% water, 0.1% formic acid) for 1 min. Peptide separation was performed at 300 nl/min with buffer A and buffer B (100% acetonitrile, 0.1% formic acid) over a linear gradient. High-Energy collisional dissociation was utilized to fragment peptide ions *via* data-dependent acquisition. Mass spectral data were processed with Proteome Discoverer (v. 2.3) and protein database search was carried out in Mascot search engine (Matrix Science, LLC; v. 2.6.0). Protein searches were conducted against the *Trichoplusia ni* protein database and the human Trio SR6-GEF1 sequence. Mascot search parameters included the following: parent peptide ion tolerance of 10.0 ppm; peptide fragment ion mass tolerance of 0.020 Da; strict trypsin fragments (enzyme cleavage after the C terminus of K or R, but not if it is followed by P); fixed modification of carbamidomethyl (C); and variable modification of phospho (S, T, Y), oxidation (M), and propioamidation (C), and deamidation (NQ). Peptide identification confidence was set at 95% confidence probability based on Mascot MOWSE score. Results were transferred to Scaffold software (Proteome Software; v. 4) for further data analysis to look at peptide abundances in reference to their start position. These were utilized to plot in a frequency distribution to determine band identity.

### Cross-linking mass spectrometry

Cross-linking experiments were performed as in Sanchez *et al.* (40) with deviations noted below. Twenty five micrograms of protein was incubated in assay buffer with 100 μM BS3 (Thermo Fisher) for 30 min on ice. The reaction was quenched by adding Tris pH 7.25 to 10 mM final concentration. Protein was then acetone precipitated and the pellet was alkylated with iodoacetamide and digested with trypsin. Peptides were desalted on a 100 μl Omix C_18_ tip (Agilent), dried, and reconstituted in 100 μl of 0.1% formic acid. Mass spectrometry was performed on an Orbitrap Exploris 480 equipped with an EasySpray nanoESI source, an EasySpray 75 μm × 15 cm C_18_ column, and a FAIMS Pro ion mobility interface coupled with an UltiMate 3000 RSLCnano system (Thermo Scientific). Each sample was analyzed at four different FAIMS compensation voltages (CV = −40 V, −50 V, −60 V, −70 V) to provide gas-phase enrichment/fractionation of cross-linked peptide ions (41). Each analysis was a separate injection (2.5 μl sample). The sample was loaded at 2% B at 600 nl/min for 35 min followed by a multisegment elution gradient to 35% B at 200 nl/min over 70 min with the remaining time used for column washing and reequilibration (buffer A: 0.1% formic acid (aq); buffer B: 0.1% formic acid in acetonitrile). Precursor ions were acquired at 120,000 resolving power, and ions with charges 3 to 8+ were isolated in the quadrupole using a 1.6 m/z unit window and dissociated by HCD at 30% NCE. Product ions were measured at 30,000 resolving power. Peak lists were generated using PAVA (in house Python app), searched with Protein Prospector v6.3.23 (42), and classified as unique residue pairs using Touchstone (an in-house R library) at SVM.score ≥1.5 corresponding to a residue pair level FDR < 0.1% and then further summarized and presented as domain-domain pairs using Touchstone. A custom database consisting of the human Trio construct and a 10× longer decoy database (11 sequences total) was used in the Prospector search, using tryptic specificity with 2-missed cleavages and tolerance of 10/25 ppm (precursor/product). DSS/BS3 cross-linking was specified.

### Bio-layer interferometry

Kinetic binding assays were performed using a ForteBio BLItz instrument. Ni-NTA biosensors were prehydrated in assay buffer for 10 min prior to the experiment. Biosensors were first measured for a baseline signal for 30 s before loading His-GEF1 (0.5 μM) or SR6-GEF1 (2 μM) in assay buffer for 5 min (concentrations were optimized for reproducible biosensor loading and signal change). Biosensors were then re-equilibrated in assay buffer for 30 s before introducing varying concentrations of Rac1 (at least four concentrations per experiment) in assay buffer for 5 min to measure association. Association curves were fit to a one phase exponential curve to obtain a k_obs_ value and these values were plotted against Rac1 concentration to calculate a K_d_ from the linear fit of this line, where the y-intercept = k_off_ and slope = k_on_ (K_d_ = k_off_/k_on_). Concentration gradients were replicated at least three times independently, and the K_d_ measurements of each interaction were compared using an unpaired *t* test. Reported values are mean ± SD.

### Measurement of GEF and SR6-GEF1 impact on cell morphology

PEI was used to transfect HEK293 cells with 0.5 to 4 μg of DNA in 6-well dishes at a density of 3 × 10^5^ cells per well. Twenty four hours after transfection, cells were trypsinized and replated at a density of 2.5 × 10^4^ cells per coverslip on fibronectin-coated coverslips (10 μg/ml fibronectin). Twenty four hours post plating, cells were fixed and stained as in Lim *et al.* (43). Cells were fixed for 5 min in 2% paraformaldehyde in cytoskeleton buffer (10 mM MES pH 6.8, 138 mM KCl, 3 mM MgCl_2_, 2 mM EGTA, 320 mM sucrose). Cells were rinsed three times in Tris Buffered Saline (TBS) (20 mM Tris pH 7.4, 150 mM NaCl) and incubated with 5 μg/ml Alexa Fluor Wheat Germ Agglutinin 555 in TBS (Thermo Fisher) for 10 min to visualize the cell membrane when imaging. Cells were washed another three times in TBS, then permeabilized for 10 min in 0.3% TritonX-100/TBS and washed another three times in 0.1% TritonX-100/TBS. Cells were blocked for 30 min in antibody dilution buffer (ADB) (0.1% TritonX-100, 2% bovine serum albumin, 0.1% NaN_3_, 10% fetal bovine serum, TBS) and incubated with primary antibody (ADB containing a 1:2000 dilution of Goat Anti-GFP, Rockland) at 4 °C overnight. The next morning, cells were washed in 0.1% TritonX-100/TBS three times and incubated in secondary antibody for 1 h at room temperature (in ADB, 1:2000 Alexa Fluor 488 Donkey Anti-Goat, Abcam). Cells were washed once in 0.1% TritonX-100/TBS, once in TBS, and then mounted onto glass slides using AquaMount (Lerner Laboratories). After drying, coverslips were sealed using clear nail polish and imaged using a 40× objective on a spinning disk confocal microscope (UltraVIEW VoX spinning disk confocal (PerkinElmer) Nikon Ti-E-Eclipse), collecting a full z-stack of images for each cell. Identical microscope settings were used between imaging samples.

After imaging cells, images were processed using Fiji/ImageJ (44) to generate a sum projection of the GFP channel for quantifying fluorescence as a proxy for total protein expression. Images were then analyzed using CellProfiler to semi-automatically detect cell edges and compute cell area (45). Cell area was normalized for protein expression on a single cell basis by dividing the total area of the cell by the total GFP fluorescence of the cell (a proxy for total protein expression). Two biological replicates were performed, with 25 to 40 cells quantified per group per replicate. Statistical significance of differences in the normalized cell area was determined using a one-way ANOVA between the GFP control and all other groups (two-tailed *p*-value < 0.05) and adjusted using Dunnett’s multiple comparisons test.

## Data availability

Data available upon request. Contact anthony.koleske@yale.edu for more information.

The limited proteolysis mass spectrometry data have been deposited to the ProteomeXchange Consortium *via* the PRIDE (46) partner repository with the dataset identifier PXD034393 (http://www.ebi.ac.uk/pride).

The cross-linking raw mass spectrometry data and peak lists are available in the massIVE repository (https://massive.ucsd.edu) with accession number: MSV000089621

Annotated spectra supporting the cross-linked identifications are published on MS-Viewer (https://msviewer.ucsf.edu/cgi-bin/msform.cgi?form=msviewer) with the following search keys:

Trio SR6-GEF1-WT: l4abvtas5a

Trio SR6-GEF1-E883D: mmmpkfzwvo

Trio SR6-GEF1-R1078Q: paout3qryt

Trio SR6-GEF1-D1368V: 7xhepmd94b

## Supporting information

This article contains supporting information (18).

## Conflict of interest

The authors declare no competing financial conflicts of interest.

## References

[1] Debant A., Serra-Pages C., Seipel K., O'Brien S., Tang M., Park S.H. (1996). The multidomain protein Trio binds the LAR transmembrane tyrosine phosphatase, contains a protein kinase domain, and has separate rac-specific and rho-specific guanine nucleotide exchange factor domains. Proc. Natl. Acad. Sci. U. S. A..

[2] Steven R., Kubiseski T.J., Zheng H., Kulkarni S., Mancillas J., Ruiz Morales A. (1998). UNC-73 activates the Rac GTPase and is required for cell and growth cone migrations in C. elegans. Cell.

[3] Penzes P., Johnson R.C., Kambampati V., Mains R.E., Eipper B.A. (2001). Distinct roles for the two Rho GDP/GTP exchange factor domains of kalirin in regulation of neurite growth and neuronal morphology. J. Neurosci..

[4] Bellanger J.M., Lazaro J.B., Diriong S., Fernandez A., Lamb N., Debant A. (1998). The two guanine nucleotide exchange factor domains of Trio link the Rac1 and the RhoA pathways *in vivo*. Oncogene.

[5] Chhatriwala M.K., Betts L., Worthylake D.K., Sondek J. (2007). The DH and PH domains of Trio coordinately engage Rho GTPases for their efficient activation. J. Mol. Biol..

[6] Rabiner C.A., Mains R.E., Eipper B.A. (2005). Kalirin: a dual rho guanine nucleotide exchange factor that is so much more than the sum of its many parts. Neuroscientist.

[7] Saito K., Tautz L., Mustelin T. (2007). The lipid-binding SEC14 domain. Biochim. Biophys. Acta.

[8] Schiller M.R., Ferraro F., Wang Y., Ma X.M., McPherson C.E., Sobota J.A. (2008). Autonomous functions for the Sec14p/spectrin-repeat region of Kalirin. Exp. Cell Res..

[9] Vishwanatha K.S., Wang Y.P., Keutmann H.T., Mains R.E., Eipper B.A. (2012). Structural organization of the nine spectrin repeats of Kalirin. Biochemistry.

[10] Bircher J.E., Koleske A.J. (2021). Trio family proteins as regulators of cell migration and morphogenesis in development and disease - mechanisms and cellular contexts. J. Cell Sci..

[11] Paskus J.D., Herring B.E., Roche K.W. (2020). Kalirin and trio: RhoGEFs in synaptic transmission, plasticity, and complex brain disorders. Trends Neurosci..

[12] Katrancha S.M., Wu Y., Zhu M., Eipper B.A., Koleske A.J., Mains R.E. (2017). Neurodevelopmental disease-associated de novo mutations and rare sequence variants affect TRIO GDP/GTP exchange factor activity. Hum. Mol. Genet..

[13] Pengelly R.J., Greville-Heygate S., Schmidt S., Seaby E.G., Jabalameli M.R., Mehta S.G. (2016). Mutations specific to the Rac-GEF domain of TRIO cause intellectual disability and microcephaly. J. Med. Genet..

[14] Sadybekov A., Tian C., Arnesano C., Katritch V., Herring B.E. (2017). An autism spectrum disorder-related de novo mutation hotspot discovered in the GEF1 domain of Trio. Nat. Commun..

[15] Barbosa S., Debant A., Schmidt S., Baralle D. (2020). Opposite modulation of RAC1 by mutations in TRIO is associated with distinct, domain-specific neurodevelopmental disorders. Am. J. Hum. Genet..

[16] Newsome T.P., Schmidt S., Dietzl G., Keleman K., Asling B., Debant A. (2000). Trio combines with dock to regulate Pak activity during photoreceptor axon pathfinding in Drosophila. Cell.

[17] Estrach S., Schmidt S., Diriong S., Penna A., Blangy A., Fort P. (2002). The human Rho-GEF trio and its target GTPase RhoG are involved in the NGF pathway, leading to neurite outgrowth. Curr. Biol..

[18] Blaise A.M., Corcoran E.E., Wattenberg E.S., Zhang Y.L., Cottrell J.R., Koleske A.J. (2022). *In vitro* fluorescence assay to measure GDP/GTP exchange of guanine nucleotide exchange factors of Rho family GTPases. Biol. Methods Protoc..

[19] Jumper J., Evans R., Pritzel A., Green T., Figurnov M., Ronneberger O. (2021). Highly accurate protein structure prediction with AlphaFold. Nature.

[20] Varadi M., Anyango S., Deshpande M., Nair S., Natassia C., Yordanova G. (2021). AlphaFold protein structure database: massively expanding the structural coverage of protein-sequence space with high-accuracy models. Nucleic Acids Res..

[21] Ward J.J., McGuffin L.J., Bryson K., Buxton B.F., Jones D.T. (2004). The DISOPRED server for the prediction of protein disorder. Bioinformatics.

[22] Hall A. (1998). Rho GTPases and the actin cytoskeleton. Science.

[23] Wells C.M., Walmsley M., Ooi S., Tybulewicz V., Ridley A.J. (2004). Rac1-deficient macrophages exhibit defects in cell spreading and membrane ruffling but not migration. J. Cell Sci..

[24] Debreceni B., Gao Y., Guo F., Zhu K., Jia B., Zheng Y. (2004). Mechanisms of guanine nucleotide exchange and Rac-mediated signaling revealed by a dominant negative trio mutant. J. Biol. Chem..

[25] McPherson C.E., Eipper B.A., Mains R.E. (2005). Multiple novel isoforms of Trio are expressed in the developing rat brain. Gene.

[26] van Rijssel J., Hoogenboezem M., Wester L., Hordijk P.L., Van Buul J.D. (2012). The N-terminal DH-PH domain of Trio induces cell spreading and migration by regulating lamellipodia dynamics in a Rac1-dependent fashion. PLoS One.

[27] Schiller M.R., Chakrabarti K., King G.F., Schiller N.I., Eipper B.A., Maciejewski M.W. (2006). Regulation of RhoGEF activity by intramolecular and intermolecular SH3 domain interactions. J. Biol. Chem..

[28] Schmidt A., Hall A. (2002). Guanine nucleotide exchange factors for rho GTPases: turning on the switch. Genes Dev..

[29] Xu Z., Gakhar L., Bain F.E., Spies M., Fuentes E.J. (2017). The Tiam1 guanine nucleotide exchange factor is auto-inhibited by its pleckstrin homology coiled-coil extension domain. J. Biol. Chem..

[30] Bellanger J.M., Estrach S., Schmidt S., Briancon-Marjollet A., Zugasti O., Fromont S. (2003). Different regulation of the Trio Dbl-homology domains by their associated PH domains. Biol. Cell.

[31] Kubiseski T.J., Culotti J., Pawson T. (2003). Functional analysis of the Caenorhabditis elegans UNC-73B PH domain demonstrates a role in activation of the Rac GTPase *in vitro* and axon guidance *in vivo*. Mol. Cell. Biol..

[32] Tian C., Paskus J.D., Fingleton E., Roche K.W., Herring B.E. (2021). Autism spectrum disorder/intellectual disability-associated mutations in trio disrupt neuroligin 1-mediated synaptogenesis. J. Neurosci..

[33] Terry-Lorenzo R.T., Torres V.I., Wagh D., Galaz J., Swanson S.K., Florens L. (2016). Trio, a rho family GEF, interacts with the presynaptic active zone proteins Piccolo and Bassoon. PLoS One.

[34] Timmerman I., Heemskerk N., Kroon J., Schaefer A., van Rijssel J., Hoogenboezem M. (2015). A local VE-cadherin and Trio-based signaling complex stabilizes endothelial junctions through Rac1. J. Cell Sci..

[35] van Rijssel J., Kroon J., Hoogenboezem M., van Alphen F.P., de Jong R.J., Kostadinova E. (2012). The Rho-guanine nucleotide exchange factor Trio controls leukocyte transendothelial migration by promoting docking structure formation. Mol. Biol. Cell.

[36] Tao T., Sun J., Peng Y., Li Y., Wang P., Chen X. (2019). Golgi-resident TRIO regulates membrane trafficking during neurite outgrowth. J. Biol. Chem..

[37] Neubrand V.E., Thomas C., Schmidt S., Debant A., Schiavo G. (2010). Kidins220/ARMS regulates Rac1-dependent neurite outgrowth by direct interaction with the RhoGEF Trio. J. Cell Sci..

[38] Son K., Smith T.C., Luna E.J. (2015). Supervillin binds the rac/rho-GEF trio and increases trio-mediated Rac1 activation. Cytoskeleton (Hoboken).

[39] Guex N., Peitsch M.C. (1997). Swiss-Model and the Swiss-pdbViewer: an environment for comparative protein modeling. Electrophoresis.

[40] Sanchez N.A., Kallweit L.M., Trnka M.J., Clemmer C.L., Al-Sady B. (2021). Heterodimerization of H3K9 histone methyltransferases G9a and GLP activates methyl reading and writing capabilities. J. Biol. Chem..

[41] Schnirch L., Nadler-Holly M., Siao S.W., Frese C.K., Viner R., Liu F. (2020). Expanding the depth and sensitivity of cross-link identification by differential ion mobility using high-field asymmetric waveform ion mobility spectrometry. Anal. Chem..

[42] Trnka M.J., Baker P.R., Robinson P.J., Burlingame A.L., Chalkley R.J. (2014). Matching cross-linked peptide spectra: only as good as the worse identification. Mol. Cell. Proteomics.

[43] Lim C., Berk J.M., Blaise A., Bircher J., Koleske A.J., Hochstrasser M. (2020). Crystal structure of a guanine nucleotide exchange factor encoded by the scrub typhus pathogen Orientia tsutsugamushi. Proc. Natl. Acad. Sci. U. S. A..

[44] Schindelin J., Arganda-Carreras I., Frise E., Kaynig V., Longair M., Pietzsch T. (2012). Fiji: an open-source platform for biological-image analysis. Nat. Methods.

[45] McQuin C., Goodman A., Chernyshev V., Kamentsky L., Cimini B.A., Karhohs K.W. (2018). CellProfiler 3.0: next-generation image processing for biology. PLoS Biol..

[46] Perez-Riverol Y., Bai J., Bandla C., Hewapathirana S., García-Seisdedos D., Kamatchinathan S. (2022). The PRIDE database resources in 2022: a hub for mass spectrometry-based proteomics evidences. Nucleic Acids Res..

